# The Spectrum of Interstitial Lung Disease Associated with Autoimmune Diseases: Data of a 3.6-Year Prospective Study from a Referral Center of Interstitial Lung Disease and Lung Transplantation †

**DOI:** 10.3390/jcm9061606

**Published:** 2020-05-26

**Authors:** Belén Atienza-Mateo, Sara Remuzgo-Martínez, Víctor Manuel Mora Cuesta, David Iturbe-Fernández, Sonia Fernández-Rozas, Diana Prieto-Peña, Mónica Calderón-Goercke, Alfonso Corrales, Gerardo Blanco Rodríguez, José Javier Gómez-Román, Miguel Ángel González-Gay, José Manuel Cifrián

**Affiliations:** 1 Research Group on Genetic Epidemiology and Atherosclerosis in Systemic Diseases and in Metabolic Bone Diseases of the Musculoskeletal System, IDIVAL, 39011 Santander, Spain; mateoatienzabelen@gmail.com (B.A.-M.); sara.r.mtz@gmail.com (S.R.-M.); victormanuel.mora@scsalud.es (V.M.M.C.); david.iturbe@scsalud.es (D.I.-F.); soniam.fernandez@scsalud.es (S.F.-R.); diana.prieto.pena@gmail.com (D.P.-P.); monipcg89@hotmail.com (M.C.-G.); afcorralesm@hotmail.com (A.C.); miguelaggay@hotmail.com (M.Á.G.-G.); 2 Department of Rheumatology, Hospital Universitario Marqués de Valdecilla, 39008 Santander, Spain; 3 Department of Pneumology, Hospital Universitario Marqués de Valdecilla, 39008 Santander, Spain; 4 Department of Radiology, Hospital Universitario Marqués de Valdecilla, 39008 Santander, Spain; gerardo.blanco@scsalud.es; 5 Department of Pathology, Hospital Universitario Marqués de Valdecilla, 39008 Santander, Spain; josejavier.gomez@scsalud.es; 6 School of Medicine, Universidad de Cantabria, 39011 Santander, Spain; 7 Cardiovascular Pathophysiology and Genomics Research Unit, School of Physiology, Faculty of Health Sciences, University of the Witwatersrand, Johannesburg 2193, South Africa

**Keywords:** interstitial lung disease, rheumatic autoimmune diseases, connective tissue diseases, interstitial pneumonia with autoimmune features

## Abstract

Interstitial lung disease (ILD) may occur in patients with a rheumatic autoimmune disease (AD), increasing their risk of morbidity and mortality. However, little is known about the prevalence of AD in patients diagnosed with an ILD. In this prospective study, we determined the spectrum of ILD associated with AD (AD-ILD) among patients sent for assessment to a single clinic of ILD and lung transplantation from a referral center between May 2016 and December 2019. ILD diagnosis was made by pneumologists based on clinical and radiological findings and pulmonary function test abnormalities. All patients with ILD were also assessed by experienced rheumatologists. During the period of assessment, 338 patients were diagnosed with ILD. Among them, 32.8% fulfilled definitions for an AD. Most cases with AD-ILD had a diagnosis of rheumatoid arthritis (27.0%), systemic sclerosis (26.1%) or anti-synthetase syndrome (17.1%). Interestingly, 18% of the patients with AD-ILD were diagnosed as having an interstitial pneumonia with autoimmune features. Antinuclear antibodies and non-specific interstitial pneumonia were the most frequent positive autoantibodies and radiological pattern found in AD-ILD patients, respectively. In conclusion, our study indicates that a high number of ILD patients have a related AD. Consequently, close collaboration among rheumatologists and pneumologists is needed.

## 1. Introduction

Interstitial lung diseases (ILDs) comprise a wide range of parenchymal lung disorders with a known possible cause (e.g., allergens, occupational exposures, pharmacotoxicity or rheumatic autoimmune diseases (ADs)) or with an unknown origin, also called idiopathic interstitial pneumonias (IIPs). The latter are classified as major, rare or unclassifiable IIPs. Idiopathic pulmonary fibrosis (IPF) is one of the major chronic fibrosing IIP [1]. An early and precise diagnosis of these ILDs is crucial since the different pathologies that encompass ILD have different prognoses and therapeutic options [2].

ADs are a compilation of pathological entities characterized by the existence of circulating autoantibodies that can produce systemic organ damage [3]. The group of rheumatic ADs mainly includes connective tissue diseases (CTD), also known as collagen vascular diseases, such as rheumatoid arthritis (RA), systemic sclerosis (SSc), systemic lupus erythematosus, primary Sjögren’s syndrome, idiopathic inflammatory myositis (IIM) (polymyositis, dermatomyositis, anti-synthetase syndrome) and mixed connective tissue disease. Systemic vasculitis and spondyloarthropathies are also incorporated in the ADs spectrum [4,5]. ILD may occur in any of these ADs, especially in the context of a CTD, and vice versa, with variable frequency and severity [6,7]. The prevalence of ILD in patients with an established AD varies from different reports and the particular AD. In this regard, it has been reported that SSc and IIM are associated more frequently with an ILD development (prevalence ranges from 35 to 53% for SSc and 20 to 86% for IIM). A clinically significant ILD is found in 8–15% of RA patients, whereas subclinical ILD detected by high-resolution computed tomography (HRCT) may occur in around 30% of RA patients [2,7,8,9,10,11]. However, little is known about the prevalence of AD in patients diagnosed with an ILD, being reported that approximately 30% of them fulfill criteria of an AD [12].

In 2015, the European Respiratory Society/American Thoracic Society proposed the term of interstitial pneumonia with autoimmune features (IPAF) to describe patients with ILD and clinical, serological, radiographic and/or histological characteristics that suggest the presence of an underlying AD but do not meet the criteria established for a CTD [13]. This concept is broadening thanks to the knowledge expansion in this area, changes in CTD criteria and identification of new biomarkers [14,15,16].

The presence of an ILD entails an increase in the patient’s morbidity and mortality, with different prognostic features depending on the subtype of ILD. Usually, the long-term prognosis of patients with AD-associated ILD (AD-ILD) is less severe than that of IPF. This may be in part due to the use of immunosuppressive therapies for the treatment of the underlying AD [7,17,18]. In addition, in some cases, patients initially diagnosed as having IPF may become an AD-ILD, which implies different therapeutic strategies to those of IPF [19]. Because of that, careful clinical and laboratory evaluation is required in patients with IPF, excluding the presence of an underlying AD, even in the absence of obvious autoimmune features. In this sense, the participation of rheumatologists becomes essential in the multidisciplinary evaluation of ILDs [20,21].

In the present study, we aimed to determine the frequency and different types of AD-ILD among patients with suspected ILD sent for assessment to a single clinic of ILD and lung transplantation from a referral center. In addition, the immunological and radiological patterns were assessed in these patients.

## 2. Materials and Methods

### 2.1. Patients

We performed an observational prospective study of a series of consecutive patients referred to the Marqués de Valdecilla University Hospital (Santander, Spain) between May 2016 and December 2019. All patients gave their informed consent for inclusion in the study. The study was conducted in accordance with the Declaration of Helsinki, and the protocol was approved by the Ethics Committee of clinical research of Cantabria, Spain (2016.092). A diagnosis of ILD was made by the pneumologists based on clinical and radiological findings and pulmonary function test abnormalities following the clinical guidelines [1,22]. A definitive histological confirmation by transbronchial biopsy, surgical lung biopsy (including lung explants biopsy) or cryobiopsy was performed by experienced pathologists according to the pneumologist consideration. Since collaboration with the rheumatologists was started, we assessed not only patients sent for lung transplant evaluation but also patients with ILD to exclude the presence of an underlying AD and/or to optimize the management of an already diagnosed rheumatic condition. In this sense, all patients underwent an accurately anamnesis and physical examination to perform a rheumatological disease screening. If there was certain suspicion for an AD-ILD, autoimmune serologies were assessed.

**Figure 1:**
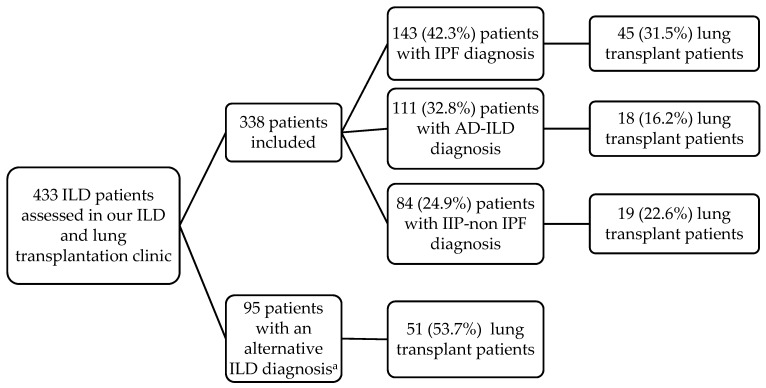
Flow diagram of patients’ assessment in our interstitial lung disease (ILD) and lung transplantation clinic. The term of “lung transplant patients” stands for patients actually transplanted by the end of follow-up of the study. ^a^ The major alternative ILD diagnosis includes hypersensitivity pneumonitis (*n* = 45), Langerhans cell histiocytosis (*n* = 14), sarcoidosis (*n* = 10) and lymphangioleiomyomatosis (*n* = 10).

A total of 433 patients satisfied the criteria for ILD. Those who fulfilled criteria for the selection of lung transplant candidates, according to the Pulmonary Transplantation Council of the International Society for Heart and Lung Transplantation [23], were included in the lung transplant list. In this regard, by the end of the study, 133 patients had received a lung transplant. Patients with hypersensitivity pneumonitis (*n* = 45), Langerhans cell histiocytosis (*n* = 14), sarcoidosis (*n* = 10), lymphangioleiomyomatosis (*n* = 10), pneumoconiosis (*n* = 6), asbestosis (*n* = 3), Hermansky–Pudlak syndrome (*n* = 3), drug and radiation-induced ILD (*n* = 3) and lipoid pneumonia (*n* = 1) were excluded to have a more homogeneous group. Finally, 338 patients with ILD were assessed and distributed in 3 categories according to their diagnosis: AD-ILD, IPF and IIP-non IPF. Different rheumatic ADs were classified according to the applicable diagnostic criteria [24,25,26,27,28,29,30]. IPAF was also considered as part of the AD-ILD spectrum [13]. Figure 1 shows the workflow of the 433 patients assessed in our clinic.

### 2.2. Methods

Demographic and clinical features were recorded in the whole group of ILD patients, including sex, age, smoking status and pulmonary function tests (PFTs). In addition, in the AD-ILD group, autoantibodies profile (rheumatoid factor (RF), anti-citrullinated protein antibody (ACPA), antinuclear antibody (ANA), anti-SSa/Ro, anti-SSb/La, anti-Scl 70 and myositis-associated antibodies, among others) and HRCT images of the chest were evaluated by experienced immunologists and radiologists, respectively. The HRCT patterns were stratified according to the histopathologic criteria for usual interstitial pneumonia (UIP) pattern of the Fleischner Society: UIP pattern, probable UIP pattern, indeterminate for UIP pattern and features most consistent with an alternative diagnosis [31].

### 2.3. Descriptive Statistical Analysis

Results were expressed as number of individuals (n) and percentage (%) for categorical variables. The differences in gender, smoking habit and lung transplantation between groups were analyzed by chi-squared test. For continuous variables, Shapiro–Wilk test was performed to determine the distribution of the data. Mean ± standard deviation or median [25th–75th interquartile range] were used when data were normally or not normally distributed, respectively. Age and PFTs between AD-ILD, IPF and IIP-non IPF patients were compared by Kruskal–Wallis and ANOVA test, respectively. In addition, differences between age and PFTs were also determined between pairwise comparison by Mann–Whitney U-test and Student’s t-test, respectively. A *p*-value <0.05 was considered statistically significant. Statistical analysis was carried out with STATA statistical software 12/SE (Stata Corp., College Station, TX, USA).

## 3. Results

### 3.1. Characteristics and Categories of ILD Patients According to Their Diagnosis

During the period of assessment (3.6 years), a total of 338 patients (108 women/230 men) diagnosed with ILD at the reference clinic mentioned above were included. The median age at ILD diagnosis was 59 (52–64) years (Table 1). The diagnosis of ILD was histologically confirmed in 170 (50.3%) patients. Among the 338 ILD patients, 143 (42.3%) were diagnosed as having IPF, 111 (32.8%) fulfilled definitions for an AD and 84 (24.9%) patients were classified as having an IIP-non IPF (49 with unclassifiable IIP, 23 with idiopathic non-specific interstitial pneumonia (NSIP), 5 with pleuroparenchymal fibroelastosis, 4 with respiratory bronchiolitis-ILD, 1 with desquamative interstitial pneumonia, 1 with idiopathic lymphoid interstitial pneumonia, and 1 with cryptogenic organizing pneumonia). Demographic and clinical characteristics of the ILD patients are shown in Table 1.

### 3.2. Spectrum of AD-ILD Patients

Regarding the group of the 111 AD-ILD patients, most patients had a diagnosis of RA (27.0%, *n* = 30), SSc (26.1%, *n* = 29) or anti-synthetase syndrome (17.1%, *n* = 19). Interestingly, 20 patients with AD-ILD (18.0%) were diagnosed as having an IPAF. Other ADs associated with ILD found at a lower frequency were primary Sjögren’s syndrome (5.4%, *n* = 6), amyopathic dermatomyositis (1.8%, *n* = 2), systemic lupus erythematosus (1.8%, *n* = 2), eosinophilic granulomatosis with polyangiitis (0.9%, *n* = 1), microscopic polyangiitis (0.9%, *n* = 1) and mixed connective tissue disease (0.9%, *n* = 1) (Table 2).

Within the serological data available, the most frequent positive autoantibodies found in AD-ILD patients were ANA (71.1%), myositis-associated antibodies (47.3%), ACPA (42.4%), RF (39.2%), anti-SSa/Ro (32.1%) and anti-Scl 70 (24.6%) (Table 2). In particular, 88.9% of RA-ILD patients were ACPA and RF positive whereas 28.6% were ANA positive. In SSc-ILD patients, the most frequent positive autoantibody was ANA (95.7%) followed by anti-Scl 70 (60.9%). All patients with anti-synthetase syndrome (*n* = 19) had positive myositis-associated antibodies, being the most frequent anti-Ro 52 (42.1%, *n* = 8/19), anti-Pl 7 (36.8%, *n* = 7/19), anti-Pl 12 (21.1%, *n* = 4/19) and anti-Jo 1 (21.1%, *n* = 4/19). Interestingly, in IPAF patients, the most frequent positive autoantibody was ANA (84.2%), followed by RF (25%).

Regarding the HRCT patterns, NSIP was the predominant radiological pattern found in patients with AD-ILD (39.1%, *n* = 43/110), whereas UIP pattern was present in 37.3% of the patients (*n* = 41/110). However, it is worth mentioning that 16% of them had a probable UIP pattern (Table 2). Specifically, NSIP was the most frequent pattern in patients with SSc-ILD and anti-synthetase syndrome (50% and 52.6%, respectively) whereas UIP was the main pattern in RA-ILD and IPAF patients (60% and 40%, respectively) (Table 3). Additionally, 34 (30.6%) of these 111 patients underwent a lung biopsy. The most common histological finding was that suggestive of UIP (35.3%, *n* = 12/34), followed by NSIP (17.6%, *n* = 6/34) and other alternative patterns (17.6%, *n* = 6/34). Description of histological findings and HRCT patterns, according to each rheumatic AD is shown in Supplementary Table S1.

## 4. Discussion

In reference centers such as ours, patients from different parts of the country are treated for lung processes that often require transplantation. In this regard, there is great concern among pneumologists and rheumatologists about the need to establish early recognition and management of a related autoimmune pathology in patients referred for an ILD. However, the confirmation of an ILD diagnosis associated with an AD requires a highly qualified multidisciplinary team [21,32].

In this 3.6-year prospective study, we determined the frequency and the spectrum of the different types of AD-ILD among 338 consecutive patients referred to our ILD multidisciplinary unit. As expected, IPF was the most frequent ILD (42.3%). Interestingly, 32.8% (*n* = 111) of the ILD patients had an associated AD. This relevant frequency is in accordance with a study reported by of Mittoo et al., where 34 of 114 patients with ILD (30%) had a well-defined CTD-ILD [12], thus reinforcing the importance to identifying this group of patients. The remaining ILD patients were diagnosed as having an IIP-non IPF (24.9%), mainly with unclassifiable IIP (14.5% of ILD patients). Similarly, it has been reported that around 15% of ILD patients have unclassifiable IIP, being essential an appropriate management of these patients [33].

According to the demographic and clinical features of our ILD patients, the median age at disease diagnosis was significantly higher in the IPF group compared to the AD-ILD and IIP-non IPF groups. Regarding gender, in the IPF group, the percentage of men was significantly predominant. However, in the AD-ILD group, sex distribution tended to be more balanced. This was probably due to the fact that ADs are usually more frequent in women [34]. More than a half of the patients in each group had ever smoked, but the higher percentage of smokers was in the IPF group. With respect to the PFTs, although no statistically significant differences were observed between AD-ILD and IPF groups, volume and diffusion parameters were higher in the AD-ILD group. In addition, DLCO was importantly reduced in the IPF group, as expected, due to the particular severity of lung involvement in that group. In accordance with this, the proportion of lung transplant patients was significantly higher in the IPF group compared to the AD-ILD group.

Within the group of AD-ILD patients, the most frequent rheumatic AD seen in our clinic was RA (27.0%), closely followed by SSc (26.1%). Although it has been reported that SSc is more frequently associated with ILD [7,11], it should also be taken into consideration that the prevalence of RA is higher than SSc (0.5–1% vs. 0.003–0.03% respectively [35,36]). The percentage of SSc-ILD in our center was also considerable. This may be explained by the increased detection of lung involvement through routine screening with pulmonary function tests in SSc patients or an early diagnosis of ILD in patients with suspicious symptoms of SSc, such as Raynaud’s phenomenon. A substantial percentage of patients in the group of AD-ILD met criteria for IPAF (18.0%). In keeping with this, Fernandes et al. has recently pointed out that a substantial number of patients with ILD present autoimmune features but do not fulfill criteria for any defined CTD [37]. It is worth mentioning that patients initially diagnosed with IPAF can meet the definitions for a well-established AD during their follow-up. That was the case of a woman of our series, who was diagnosed as having SSc after the performance of a nailfold videocapillaroscopy and a new immunoblot. However, IPAF’s tendency to evolve into a definitive CTD is controversial [38,39].

Within the serological available data in the AD-ILD patients from our series, ANA was the most common antibody. According to other studies, the presence of ANA in a patient with ILD, in whom an AD is suspected, has a high positive predictive value for the diagnosis of a rheumatologic condition [40]. The majority of RA-ILD patients from our series were ACPA and RF positive. These findings are in agreement with former studies that emphasized the relevance of ACPA positivity as a risk factor for the development of ILD in patients with RA [10,41,42]. More than a half of the patients with SSc-ILD showed Scl 70 antibody positivity. This frequency was higher than in former reports [43,44,45]. Therefore, our data support the claim that the presence of anti-Scl 70 in patients with SSc is an important reason for monitoring lung involvement in these patients [8,46]. All patients with anti-synthetase syndrome had positive myositis-associated antibodies, the most frequent being, in decreasing order, anti-Ro 52, anti-Pl 7, anti-Pl 12 and anti-Jo 1. These serological data are consistent with previous reports that showed a higher frequency of ILD in anti-Jo-1 negative anti-synthetase syndrome patients [47,48,49]. As previously reported, patients from our series with diagnosis of IPAF presented high positivity of ANA antibodies [32,37].

In accordance with previous reports, HRCT images in the group of AD-ILD patients from our series yielded a predominant NSIP pattern [1,50]. However, an UIP pattern was also common among AD-ILD patients. This may be explained by the high number of patients with RA diagnosis in our study. It also highlights the need of excluding an underlying AD in patients with UIP findings [51,52]. Lung histological study was performed in a few patients with AD-ILD. Consistently with Mittoo et al., a histology suggestive of UIP was found in a considerable number of patients, demonstrating again that UIP does not exclude the possibility of an associated AD [12]. 

Our findings might be somehow limited due to the fact the autoimmune screening was addressed to patients with certain clinical suspicion for an AD-ILD. Consequently, the number of IPAF patients could have been underestimated. Otherwise, the major strength of this study was the prospective data collection of a high number of ILD patients, reflecting what is happening in real clinical practice. In addition, the prevalence of AD-ILD among patients with suspected ILD has not been widely reported before. It would also be interesting to prospectively assess the outcomes of these AD-ILD patients in the future, especially the predictors of response to different therapies. In this line, further studies are needed to report the evolution of AD-ILD patients undergoing lung transplantation.

In conclusion, our study indicates that a high number of patients seen in the ILD clinics have a related AD. Consequently, close collaboration among rheumatologists and pneumologists is needed to make an accurate diagnosis that may facilitate immune therapy and a better patient outcome.

## Figures and Tables

**Table 1:** Demographic and clinical features of 338 patients with ILD included in this study.

	ILD Patients, *n* = 338	AD-ILD, *n* = 111	IPF, *n* = 143	IIP-Non IPF, *n* = 84	*p*-Value ^a^	*p*-Value ^b^	*p*-Value ^c^	*p*-Value ^d^
Sex (women/men), *n* (%)	108/230 (32.0/68.0)	53/58 (47.7/52.3)	24/119 (16.8/83.2)	31/53 (36.9/63.1)	<0.001	<0.001	0.13	<0.001
Age at ILD diagnosis, years, median [IQR]	59 (52–64)	57 (50–63)	60 (55–64)	58 (44–66)	0.01	0.006	0.87	0.025
Smoking ever, *n* (%)	232 (68.6)	69 (62.2)	113 (79.0)	50 (59.5)	0.002	0.003	0.71	0.002
Pulmonary function tests								
FEV1 (% predicted), mean ± SD	76.4 ± 22.6	79.2 ± 24.7	77.5 ± 20.3	70.8 ± 22.8	0.029	0.56	0.018	0.024
FVC (% predicted), mean ± SD	77.6 ± 23.0	81.8 ± 25.0	77.4 ± 19.9	72.4 ± 24.3	0.018	0.12	0.009	0.09
FEV1/FVC (% predicted), mean ± SD	78.9 ± 9.6	77.9 ± 8.7	79.0 ± 9.2	80.0 ± 11.2	0.33	0.35	0.15	0.47
DLCO (% predicted) mean ± SD	36.7 ± 15.1	38.2 ± 14.7	34.9 ± 15.4	37.9 ± 14.9	0.28	0.15	0.92	0.25
Actual lung transplant received, *n* (%)	82 (24.3)	18 (16.2)	45 (31.5)	19 (22.6)	0.018	0.005	0.26	0.15

AD: autoimmune diseases, DLCO: diffusing capacity of the lung for carbon monoxide, FEV1: forced expiratory volume in one second, FVC: forced vital capacity, IIP: idiopathic interstitial pneumonia, ILD: interstitial lung disease, IPF: idiopathic pulmonary fibrosis, IQR: interquartile range, SD: standard deviation. ^a^ Comparison between AD-ILD, IPF and IIP-non IPF patients. ^b^ Comparison between AD-ILD and IPF patients. ^c^ Comparison between AD-ILD and IIP-non IPF patients. ^d^ Comparison between IPF and IIP-non IPF patients.

**Table 2:** Spectrum of the 111 AD-ILD patients included in the study.

**Rheumatic autoimmune disease**	***n* (%)**
Rheumatoid arthritis	30 (27.0)
Systemic sclerosis	29 (26.1)
Interstitial pneumonia with autoimmune features	20 (18.0)
Anti-synthetase syndrome	19 (17.1)
Others ^a^	13 (11.8)
**Autoantibody profile ^b^**	***n/N* (%)**
Rheumatoid factor	31/79 (39.2)
Anti–citrullinated protein antibody	25/59 (42.4)
Antinuclear antibody	64/90 (71.1)
Anti-SSa (Ro)	17/53 (32.1)
Anti-SSb (La)	4/50 (8.0)
Anti-Scl 70	15/61 (24.6)
Myositis-associated antibodies ^c^	26/56 (46.4)
Others ^d^	15/67 (22.4)
**High-resolution computed tomography pattern**	***n* (%)**
UIP pattern	41 (37.3)
Probable UIP pattern	16 (14.5)
Indeterminate for UIP pattern	3 (2.7)
Features most consistent with an alternative diagnosis	
NSIP pattern	43 (39.1)
Non-NSIP pattern	7 (6.4)

AD: autoimmune diseases, ILD: interstitial lung disease, N: data available, NSIP: non-specific interstitial pneumonia, UIP: usual interstitial pneumonia. ^a^ Includes primary Sjögren’s syndrome (*n* = 6), amyopathic dermatomyositis (*n* = 2), systemic lupus erythematosus (*n* = 2), eosinophilic granulomatosis with polyangiitis (*n* = 1), microscopic polyangiitis (*n* = 1) and mixed connective tissue disease (*n* = 1). ^b^ Patients may have more than one antibody. ^c^ Includes anti-Ro 52 (*n* = 10), anti-Pl 7 (*n* = 7), anti-Pl 12 (*n* = 4), anti-Jo 1 (*n* = 4), anti-PML-Scl (*n* = 2), anti-Ku (*n* = 2), anti-MDA 5 (*n* = 1), anti-NOR 90 (*n* = 1) and anti-fibrillarin (*n* = 1) antibodies. ^d^ Includes anti-RNP (*n* = 5), anti-phospholipid (*n* = 4), anti-neutrophil cytoplasmic (*n* = 4), anti-DNA (*n* = 1), anti-smooth muscle (*n* = 1) and anti-Smith (*n* = 1) antibodies.

**Table 3:** Relation between HRCT pattern and the main AD-ILDs of this study.

	RA-ILD *n* (%)	SSc-ILD *n* (%)	IPAF *n* (%)	Anti-Synthetase Syndrome *n* (%)
UIP pattern	18 (60.0)	6 (21.5)	8 (40.0)	5 (26.3)
Probable UIP pattern	2 (6.7)	4 (14.3)	4 (20.0)	4 (21.1)
Indeterminate for UIP pattern	1 (3.3)	2 (7.1)	-	-
Features most consistent with an alternative diagnosis				
NSIP pattern	8 (26.7)	14 (50.0)	5 (25.0)	10 (52.6)
Non-NSIP pattern	1 (3.3)	2 (7.1)	3 (15.0)	-

AD: autoimmune diseases, HRCT: high-resolution computed tomography, ILD: interstitial lung disease, IPAF: interstitial pneumonia with autoimmune features, NSIP: non-specific interstitial pneumonia, RA: rheumatoid arthritis, SSc: systemic sclerosis, UIP: usual interstitial pneumonia. Non information about HRCT pattern was available in one patient with SSc-ILD.

## Supplementary Materials

The following are available online at https://www.mdpi.com/2077-0383/9/6/1606/s1, Table S1: Description of histological and radiological findings in 34 patients of the AD-ILD group.
