# Supplementary material for: The Spectrum of Interstitial Lung Disease Associated with Autoimmune Diseases: Data of a 3.6-Year Prospective Study from a Referral Center of Interstitial Lung Disease and Lung Transplantation
Source: J Clin Med. 2020 May 26;9(6):1606. doi: 10.3390/jcm9061606 (PMC7356573; doi:10.3390/jcm9061606)
Supplement: Supplementary file 1 [file jcm-09-01606-s001.pdf]

Supplementary Materials: The Spectrum of Interstitial Lung Disease Associated with Autoimmune Diseases: Data of a 3.6-Year Prospective Study from a Referral Center of Interstitial Lung Disease and Lung Transplantation †

Table S1. Description of histological and radiological findings in 34 patients of the AD-ILD group.

**Rheumatic AD (n)**	**Histological finding (n)**	**HRCT pattern (n)**
Anti-synthetase syndrome (8)	NSIP (2)<br>Non-classifiable fibrosis (2)<br>Non-diagnostic (2)<br>UIP (1)<br>Other (1)	NSIP pattern (7)<br>UIP pattern (1)
Interstitial pneumonia with autoimmune features (8)	UIP (4)<br>Non-classifiable fibrosis (2)<br>NSIP (1)<br>Other (1)	NSIP pattern (4)<br>UIP pattern (3)<br>Probable UIP pattern (1)
Rheumatoid arthritis (7)	UIP (4)<br>Non-classifiable fibrosis (1)<br>NSIP (1)<br>Non-diagnostic (1)	UIP pattern (3)<br>NSIP pattern (3)<br>Probable UIP pattern (1)
Systemic sclerosis (6)	UIP (2)<br>NSIP (2)<br>Not available (2)	NSIP pattern (3)<br>UIP pattern (1)<br>Indeterminate for UIP pattern (1)<br>Not available (1)
Primary Sjögren's syndrome (2)	UIP (1)<br>Other (1)	UIP pattern (1)<br>NSIP pattern (1)

Amyopathic dermatomyositis (1)	Other (1)	NSIP pattern (1)
Systemic lupus erythematosus (1)	Other (1)	Probable UIP pattern (1)
Eosinophilic granulomatosis with polyangiitis (1)	Other (1)	Probable UIP pattern (1)

AD: autoimmune diseases, HRCT: high-resolution computed tomography pattern, ILD: interstitial lung disease, NSIP: non-specific interstitial pneumonia, UIP: usual interstitial pneumonia.

Other includes: diffuse alveolar damage, respiratory bronchiolitis, organizing pneumonia, vasculitis.
